# Balancing Privacy, Trust, and Equity: Patient Perspectives on Substance Use Disorder Data Sharing

**DOI:** 10.3390/ijerph22040617

**Published:** 2025-04-15

**Authors:** Mengyi Wei, Michael Todd, Aimee N. C. Campbell, Darwyn Chern, Eric Lott, Mary J. Whitfield, Nick Stavros, Elise Greenberg, Adela Grando

**Affiliations:** 1 College of Health Solutions, Arizona State University, Phoenix, AZ 85004, USA; mwei32@asu.edu; 2 Edson College of Nursing and Health Innovation, Arizona State University, Phoenix, AZ 85004, USA; mike.todd@asu.edu; 3 Department of Psychiatry, Columbia University Irving Medical Center, New York, NY 10032, USA; anc2002@cumc.columbia.edu; 4 Copa Health, Mesa, AZ 85205, USA; darwyn.chern@copahealth.org (D.C.); rivkagreen@gmail.com (E.G.); 5 Community Bridges, Phoenix, AZ 85034, USA; elott@cbridges.com; 6 Jewish Family and Children’s Services, Phoenix, AZ 85037, USA; maryjo.whitfield@jfcsaz.org; 7 Community Medical Services, Phoenix, AZ 85021, USA; nick.stavros@cmsgiveshope.com

**Keywords:** substance use disorder, data sharing, stigma, willingness to share, health equity

## Abstract

Background: Sharing substance use disorder (SUD) data is essential for advancing equitable healthcare and improving outcomes for marginalized populations. However, concerns about privacy, stigma, and adherence to data privacy regulations often hinder effective data sharing. This study explores patient preferences and considerations related to sharing SUD-related medical records, with a focus on the sociocultural and systemic factors that shape their willingness to share. Methods: A total of 357 adult patients from four community-based clinics in Arizona participated in a cross-sectional electronic survey. The survey assessed sociodemographic factors, experiences of stigma (self-directed, anticipated, and provider-based), trust in healthcare providers, satisfaction with care, and willingness to share SUD data across various scenarios. Data were analyzed using descriptive statistics, Pearson correlations, and one-way ANOVA to uncover key associations. Results: Patients identified SUD history, diagnoses, and treatment information as particularly sensitive. Stigma was significantly correlated with increased sensitivity and reduced willingness to share data, especially with providers outside their primary facility (*p* < 0.001). In contrast, trust in providers and higher satisfaction with care were linked to greater willingness to share data with all providers (*p* < 0.01). Patients were more inclined to share SUD data during emergencies or for direct treatment purposes than for administrative or research applications (*p* < 0.001). Discussion: These findings underscore the ethical imperative to address stigma and foster trust to promote equitable SUD data sharing. Policies must empower patients with control over sensitive health information while ensuring cultural competence and fairness in care delivery. Ensuring that patients feel confident in how their data are used may encourage greater participation in health information exchange, ultimately supporting more effective and individualized SUD care.

## 1. Introduction

Substance use disorder (SUD) is a mental health condition that impairs cognitive function and behavior, leading to challenges in regulating the use of substances such as legal and illegal drugs, alcohol, and prescription medications [1]. SUD disproportionately impacts marginalized populations, exacerbating physical and mental health inequities, including heightened risks for infectious diseases, mental health disorders, overdose deaths, and chronic illnesses [1,2]. These disparities underscore the urgent need for ethical, culturally sensitive, and equity-focused approaches to SUD treatment and data management.

Efforts to integrate SUD services across the healthcare system—spanning primary care, emergency departments, mental health clinics, and inpatient hospital settings—are critical for improving access to care [1,3]. However, integration efforts are incomplete without addressing the ethical challenges of SUD data sharing. Effective data sharing is vital for enhancing care coordination, tailoring treatments, and ensuring patient safety [4]. Yet, concerns about privacy, stigma, and discriminatory practices create significant barriers, particularly for communities that already face systemic inequities in healthcare [5].

The enactment of Title 42 Code of Federal Regulations Part 2 (42 CFR Part 2, or “Part 2”) in 1975 was a pivotal moment in protecting the confidentiality of SUD treatment records [6]. This regulation sought to mitigate stigma and encourage treatment by safeguarding sensitive information [7]. However, stigma—both internalized and external—remains a significant barrier. Individuals with SUD often experience self-stigma, internalizing negative stereotypes that lead to diminished self-esteem and reluctance to seek care [5,8]. Social stigma, including negative judgments from peers, family, and even healthcare providers, compounds this issue [9,10]. Fear of provider-based discrimination further deters individuals from sharing critical health information, raising concerns about the potential for compromised care [11].

The provider–patient relationship may also affect the person’s willingness to share SUD information [12]. A positive and trusting patient–provider relationship, characterized by confidence in the secure handling of personal data, significantly increases patients’ willingness to share their information [13]. Studies have found that the quality of the patient–provider relationship plays a crucial role in influencing patients’ willingness to disclose substance use during screening processes [12,14]. Concerns over privacy and discrimination can lead to a patient being reluctant to share crucial information, often due to a lack of trust in or fear of judgment from the healthcare provider [15].

Healthcare providers want access to SUD medical records to allow them to provide safe care, make informed decisions, tailor treatments, and prevent errors [16]. However, because healthcare providers found it hard to balance the imperative to meet their patients’ need for care with compliance with Part 2 regulations, many advocated for modifications to Part 2 that would decrease the burden of compliance and facilitate sharing of patient data to improve care [17,18,19].

Empirical research on patient perspectives to inform updates and refinements of Part 2 is still limited. To date, the literature on SUD data privacy and sharing consists largely of opinion-based or legal analyses, with only a few studies examining associations among sociodemographic factors, stigma, SUD treatment efficacy, and SUD data sharing [19].

The National Institute on Drug Abuse (NIDA) funded this project, which has the overarching aim of amplifying the voices of patients and providers regarding the sharing of SUD health records. Here, we report on a study focused on patients’ preferences for granular privacy controls and their willingness to share SUD-related medical records under various scenarios when providers expressed the need for SUD data access.

This study extends prior research focused on data privacy among health behavior patients [19,20] and seeks to address that knowledge gap by exploring patient perspectives on SUD data sharing, guided by three research questions:

1. How do sociodemographic factors influence patients’ stigma, perceived sensitivity of sharing SUD data, and willingness to share SUD data?
  1a Does gender moderate the relationship between sociodemographic factors (race, income, education, and age) and key outcomes?
  1b What is the structural validity of the adapted stigma measures for SUD patients?
2. What is the relationship between the perceived sensitivity of SUD data and patients’ willingness to share SUD data?
  2a Does stigma influence patients’ perceived sensitivity of sharing SUD data?
3. What are the relationships between multidimensional factors and patients’ willingness to share SUD data?
  3a Is stigma (self-stigma, anticipated stigma, and provider-based stigma) linked with patients’ willingness to share SUD data?
  3b Is provider discrimination experience correlated with patients’ willingness to share SUD data?
  3c Are trust in providers and satisfaction with care correlated with patients’ willingness to disclose SUD-related medical records?
  3d Do providers “need to know” influence patients’ willingness to disclose SUD data?

## 2. Methods

### 2.1. Study Design

We used a cross-sectional design with self-report questionnaires administered via an online data collection platform. We recruited patient participants from four healthcare organizations serving in Arizona.

### 2.2. Patient Recruitment

We employed a convenience sampling method to recruit participants from four community-based healthcare facilities that provide SUD care. Study inclusion criteria were (a) English as the preferred language, (b) 18 years of age or older, (c) experienced substance use, and (d) clients at the participating facilities. We recruited participants through messages outlining the study’s purpose and including a participation link. Site supervisors distributed these messages via text messages, emails, and client portals.

Interested individuals accessed the participation link to review the eligibility criteria. To proceed, participants were required to self-identify as meeting all three eligibility criteria. Those who did not meet the criteria were automatically logged out of the system and restricted from accessing the survey questions, ensuring that only eligible individuals participated.

Informed consent was obtained electronically through the survey platform. Eligible participants were presented with a digital consent form explaining the study’s purpose, voluntary nature, and confidentiality measures. The consent form explicitly stated that no personal identifiers, such as names, would be collected, ensuring anonymity. Participants had to confirm their consent before proceeding to the survey questions. The Arizona State University Institutional Review Board (STUDYID 00016171) approved this study, ensuring compliance with ethical research standards.

### 2.3. Questionnaire Design

For the pilot testing of the SUD survey, we involved fourteen members of the research team and four clinical sites with expertise in medicine, clinical psychiatry, social work, SUD research, population and public health, health services administration, behavioral health services, and informatics. The research team first reviewed the survey for relevance and structural coherence, ensuring each question aligned with our study’s objectives.

To maintain neutrality and objectivity, the reviewers systematically evaluated each item for potential bias, leading language, or implicit assumptions that could influence responses. Items flagged for suggestive wording were revised or reworded based on expert feedback to eliminate unintended bias and ensure that participants could respond freely. Following this, collaborators assessed language clarity and accessibility, ensuring that the wording was easily understandable and resonated with the participants’ experiences.

Feedback from both the research team and collaborators was systematically incorporated to strike a balance between the survey’s academic rigor and participant accessibility.

We implemented a 13-question survey (see Supplementary File S1) to assess key study constructs. Each question represented either a single question or a scale containing multiple items designed to measure different dimensions of a specific construct, as follows:

1. Sociodemographic information: Six questions were related to age, gender, race/ethnicity, education level, and annual income.
2. Stigma of Substance Use: Eighteen items assessed substance use-related self-stigma, anticipated stigma, and provider-based stigma. Six items measuring self-stigma and nine items assessing anticipated stigma were selected from the Stigma and Self-Stigma Scales (SASS), originally validated for mental health settings [21]. The Cronbach’s Alpha coefficient for the self-stigma scale was 0.860, indicating good internal reliability. The anticipated stigma scale demonstrated excellent internal reliability, with a Cronbach’s Alpha coefficient of 0.921. To capture provider-based stigma, we designed three items to evaluate the patient’s comfort level in discussing their disorder, their willingness to seek help, their concerns about potential judgment or bias from healthcare providers. The 3-item provider-based stigma scale demonstrated excellent internal consistency, with a Cronbach’s Alpha coefficient of 0.929. We measured all items on a 5-point Likert scale, ranging from 1 (strongly agree) to 5 (strongly disagree). To ensure the structural validity of the adapted SASS scale for stigma and self-stigma items, we conducted both exploratory factor analysis (EFA) and confirmatory factor analysis (CFA).
3. Provider discrimination experience: We assessed participants’ experiences of healthcare provider discrimination (1) within and (2) outside the facility using a single item with a dichotomous (“yes”, “no”) response scale.
4. Perceived sensitivity of sharing substance use-related data: We used five items to assess the degree of sensitivity of individuals correlated with different types of SUD data: (1) substance use history, (2) diagnoses, (3) medications, (4) treatment protocols, and (5) results of laboratory tests. For all five items, we used a 7-point Likert-type response scale ranging from 1 (extremely sensitive) to 7 (not at all sensitive). The perceived sensitivity of the SUD data scale demonstrated excellent internal consistency, with a Cronbach’s Alpha coefficient of 0.949.
5. Patient satisfaction with care: We adapted six items measuring satisfaction from the validated Client Satisfaction Questionnaire-8 (CSQ-8), which has been used within substance use settings [22]. For all items, we used a 5-point Likert scale, ranging from 1 (strongly agree) to 5 (strongly disagree). The scale demonstrated excellent internal consistency, with a Cronbach’s Alpha coefficient of 0.963.
6. Patient trust in providers: We used five items to measure patient trust in providers. We adapted two items from the validated Client Satisfaction Questionnaire-8 (CSQ-8) [22] and added three new items. For all items, we used a 5-point Likert scale, ranging from 1 (strongly agree) to 5 (strongly disagree). The scale demonstrated good internal consistency, with a Cronbach’s Alpha coefficient of 0.823.
7. Willingness to share SUD data depending on data recipient: We used three items to assess an individual’s willingness to share SUD data with different healthcare providers: (1) provider at the facility, (2) providers outside the facility, and (3) emergency providers. We measured all items on a 5-point Likert scale, ranging from 1 (always share) to 5 (never share). The willingness to share with different providers scale demonstrated acceptable internal consistency, with a Cronbach’s Alpha coefficient of 0.717.
8. Willingness to share SUD data depending on data-sharing purpose and providers’ “need to know”: We used seven items to assess the individual’s willingness to share SUD data when there is a “need to know” scenario outside of the facility related to (1) changes to medication, (2) changes to non-pharmacologic treatments, (3) improving care, (4) research purposes, (5) emergency situations, (6) employment discussions, and (7) insurance matters (e.g., reimbursements). We measured all items on a 5-point Likert scale, ranging from 1 (always share) to 5 (never share). We combined the first five items to represent “need to know” scenarios specifically involving outside and emergency providers.

### 2.4. Data Collection

We collected survey data using Qualtrics [23]. To ensure strict anonymity and confidentiality, we did not collect participant names at any stage of the data collection process. Prior to administering the survey, we obtained written consent from all participants. We compensated participants with a 20 USD e-gift card.

### 2.5. Data Analysis

We employed descriptive statistics (i.e., means, frequencies) to summarize key characteristics of participants and their survey responses. We used Pearson correlations to examine bivariate correlations among key outcomes, including stigma, satisfaction with care, trust in providers, perceived data sensitivity, and willingness to share SUD data. To assess whether patient trust or satisfaction moderated the relationship between stigma (self-stigma, anticipated stigma, and provider-based stigma) and willingness to share SUD data, we conducted univariate ANOVAs with interaction terms. We conducted one-way analyses of variance (ANOVAs) to examine bivariate correlations of sociodemographic factors (age group, gender, race, education level, and annual income) with each outcome. Where significant differences were identified, we conducted post hoc Tukey’s Honestly Significant Difference (HSD) tests to further clarify group differences. Tukey’s HSD was chosen for its ability to compare all possible pairwise group differences. We performed all statistical analyses using SPSS 29.0 [24]. 

Additionally, sample size determination was conducted using G*Power to ensure adequate statistical power for linear and logistic regression models. A total of *n* = 300 respondents provided 80% power to detect modest associations (R^2^ ≥ 0.028, d = 0.34) in sensitivity ratings, differences between providers or purpose categories (+0.085 to +0.120), and odds ratios (1.55–1.80) for overall willingness to share. The sample size also afforded a power to detect differences of 0.20 in willingness to share across provider and purpose categories.

Finally, we used exploratory factor analysis (EFA) and confirmatory factor analysis (CFA) to examine the structure of the adapted stigma measures. EFA was conducted on both the full 15-item adapted measure and a 10-item subset that aligns with the original SASS items. CFA was performed to test a two-factor solution representing anticipated stigma and self-stigma. The CFA model was evaluated using Comparative Fit Index (CFI), Tucker–Lewis Index (TLI), and Root Mean Square Error of Approximation (RMSEA). Docksey et al.’s original CFA findings lacked key methodological details, such as whether cross-loadings were examined, co-variation of measurement errors was permitted, or full inter-factor correlations were reported [20]. These limitations restrict direct comparisons between the original SASS and our adapted measures. Therefore, we reported model fit, factor loadings, and composite reliability to assess our measures’ validity within this clinical SUD population.

To ensure consistent interpretation across constructs, all Likert scale items and composite measures were reverse coded so that higher scores reflected greater levels of the underlying construct. For example, higher scores on the willingness to share items indicated greater willingness to share SUD data, while higher scores on the stigma, trust, and satisfaction scales represented greater perceived stigma, higher trust in providers, and greater satisfaction with care, respectively.

## 3. Results

### 3.1. Demographics

We collected survey data from 380 participants who met the study inclusion criteria, and of these, 357 (93.9%) completed all the items. We excluded data from participants with incomplete surveys (*n* = 23). The final sample size exceeded the required threshold, ensuring adequate statistical power for the analyses.

Table 1 summarizes participant demographic and background characteristics. Participants ranged in age from 20 years to 81 years old (*M* = 41.7, *SD* = 11.19). For further analysis, we divided the participants into five age groups: 18–29 years (*n* = 53), 30–39 years (*n* = 107), 40–49 years (*n* = 111), 50–59 years (*n* = 66), and 60 years and older (*n* = 20). Participants were mostly men (56.0%), with more than half of the participants identifying as White (57.4%), followed by Hispanic or Latino (26.1%), and smaller proportions identifying as either Black or African American (8.7%), Native American, or Alaskan Native (3.1%). In terms of maternal nativity, most were U.S.-born (90.5%). About a quarter of the sample (28.3%) were high school graduates, with almost one-third reporting attending some college (30.8%). Over half of the participants had been in treatment for over a year (58.3%).

### 3.2. Sociodemographic Differences in Stigma, Patient Trust, and Willingness to Share SUD Data

#### 3.2.1. Gender

Provider-Based Stigma: A one-way ANOVA (Table 2 found a significant effect of gender on anticipated stigma (F(2, 354) = 2.812, *p* = 0.048). Post hoc comparisons revealed that females reported significantly higher anticipated stigma than males (*p* = 0.048). However, no significant differences were observed between the “Other” gender category and either males or females. The effect size was small, with gender accounting for 1.6% of the variance in anticipated stigma (η^2^ = 0.016, 95% CI [0.000, 0.047]).

Willingness to Share SUD Data with Providers at the Facility: Gender also influenced willingness to share SUD data at the facility (F(2, 354) = 6.729, *p* = 0.001) (Table 2). Post hoc comparisons indicated that individuals in the “Other” gender category reported significantly higher willingness to share SUD data at the facility compared to both males (*p* = 0.006) and females (*p* = 0.021). The difference between males and females approached significance (*p* = 0.061). The effect size for this difference was small to moderate, accounting for 3.7% of the variance (η^2^ = 0.037, 95% CI [0.006, 0.079]).

No significant gender-related differences were observed for self-stigma, physician stigma, satisfaction with care, patient trust in providers, or willingness to share SUD data with providers outside the facility or with emergency care providers (*p* > 0.05).

#### 3.2.2. Age

Provider-Based Stigma: A one-way ANOVA (Table 2) revealed significant age-related differences in provider-based stigma (F(4, 352) = 3.423, *p* = 0.009). Post hoc comparisons indicated that patients aged 50–59 years reported significantly higher physician stigma compared to those aged 30–39 years (*p* = 0.028) and 40–49 years (*p* = 0.047). The effect size was small, with age accounting for 0.9% of the variance in physician stigma (η^2^ = 0.009, 95% CI [0.000, 0.034]).

No significant age-related differences were observed for self-stigma, anticipated stigma, satisfaction with care, patient trust in providers, perceived sensitivity of SUD data, or willingness to share SUD data with all providers (*p* > 0.05).

#### 3.2.3. Education Level

Provider-Based Stigma: A one-way ANOVA (Table 2) revealed significant differences in physician stigma based on education level (F(6, 350) = 2.197, *p* = 0.043). Post hoc comparisons indicated that patients with a bachelor’s degree reported significantly lower physician stigma than those with some high school education (*p* = 0.020) and some college education (*p* = 0.047). The effect size was small to moderate, with 3.7% of the variance in physician stigma explained by education level (η^2^ = 0.037, 95% CI [0.003, 0.074]).

Patient Trust in Providers: Significant differences were also found for patient trust in providers (F(6, 350) = 2.829, *p* = 0.011) (Table 2). Post hoc comparisons showed that patients with some high school education reported significantly higher trust in providers compared to those with some college education (*p* = 0.036) and those with an associate’s degree (*p* = 0.033). The effect size was small, with education level accounting for 1.1% of the variance (η^2^ = 0.011, 95% CI [0.000, 0.036]).

Willingness to Share SUD Data with Providers Outside the Facility: A one-way ANOVA (Table 2) revealed significant effects for willingness to share SUD data outside the facility (F(6, 350) = 2.443, *p* = 0.025). Post hoc comparisons revealed patients with some high school education reported significantly greater willingness to share SUD data outside the facility compared to those with a bachelor’s degree (*p* = 0.031). The effect size for willingness to share SUD data outside the facility was small, accounting for 1.7% of the variance (η^2^ = 0.017, 95% CI [0.000, 0.047]).

Willingness to Share SUD Data with Emergency Care Providers: A one-way ANOVA (Table 2) revealed significant effects for willingness to share SUD data with emergency care providers (F(6, 350) = 2.977, *p* = 0.008). Post hoc comparisons revealed that patients with some high school education also reported significantly higher willingness to share SUD data with emergency care providers compared to those with a bachelor’s degree (*p* = 0.019) and those with some college education (*p* = 0.046). The effect size for willingness to share SUD data with emergency care providers was small, explaining 2.2% of the variance (η^2^ = 0.022, 95% CI [0.002, 0.054]).

No significant education-related differences were observed for self-stigma, anticipated stigma, satisfaction with care, perceived sensitivity of SUD data (*p* > 0.05), or willingness to share SUD data with providers at the facility (*p* > 0.05).

**Table 2:** Bivariate Correlations of Willingness to Share SUD Data with Perceived Sensitivity of Sharing All Types of SUD Data (*n* = 357).

Variables	Willingness Within	Willingness Outside	Willingness Emergency	Perceived Sensitivity	History Sensitivity	Diagnoses Sensitivity	Medication Sensitivity	Treatment Sensitivity	Lab Tests Sensitivity
Willingness Within	-								
Willingness Facility	0.44 **	-							
Willingness Emergency	0.50 **	0.46 **	-						
Perceived Sensitivity	0.00	−0.13 *	−0.04	-					
History Sensitivity	0.02	−0.10	0.00	0.91 **	-				
Diagnoses Sensitivity	−0.01	−0.13 *	−0.02	0.93 **	0.87 **	-			
Medication Sensitivity	0.04	−0.09	−0.06	0.92 **	0.76 **	0.81 **	-		
Treatment Sensitivity	0.03	−0.12 *	−0.05	0.94 **	0.79 **	0.83 **	0.89 **	-	
Lab Tests Sensitivity	−0.05	−0.13 *	−0.06	0.87 **	0.74 **	0.75 **	0.71 **	0.76 **	-
Mean	4.15	3.18	3.83	5.52	5.70	5.58	5.43	5.44	5.46
SD	1.12	1.34	1.35	1.64	1.64	1.76	1.86	1.44	1.88

Correlation is significant at the 0.01 level. **. Correlation is significant at the 0.05 level. *.

#### 3.2.4. Income Level

No significant differences were found based on income level for any of the measured variables, including stigma, trust, satisfaction, and willingness to share SUD data with all providers (*p* > 0.05).

#### 3.2.5. Gender Moderation of Sociodemographic Factors and Outcomes

ANOVA analysis (Table 3) results indicated that gender moderates the relationship between race and trust in providers (F(11, 341) = 1.92, *p* = 0.04), as well as willingness to share SUD data within the facility (F(11, 341) = 2.07, *p* = 0.02) and in emergency settings (F(11, 341) = 2.22, *p* = 0.01). Specifically, women reported higher trust in providers compared to men. Additionally, they showed greater willingness to share SUD data with both facility-based providers and emergency care providers than men. In contrast, gender did not significantly moderate the relationship between income, education, or age and any of the outcome variables (ps > 0.05) (Table 3).

#### 3.2.6. Structural Validity and Reliability of the Adapted Stigma Measures

Exploratory factor analysis (EFA) supported the plausibility of a two-factor solution for both item sets. Confirmatory factor analysis (CFA) results further reinforced this structure, aligning with the model reported by Docksey et al. [20] Specifically, the full item set demonstrated strong model fit (CFI = 0.998, TLI = 0.997, RMSEA = 0.049 [90% CI = 0.037–0.061]), as did the common item subset (CFI = 0.997, TLI = 0.996, RMSEA = 0.055 [90% CI = 0.037–0.074]). Standardized factor loadings ranged from 0.61 to 0.86 for the full item set and 0.64 to 0.84 for the common item subset. Composite reliability values were high for both anticipated stigma and self-stigma (full item set: 0.94 and 0.88, respectively; common item set: 0.87 and 0.88). Overall, these psychometric properties indicate that the adapted measures exhibit structural validity that is comparable or superior to the original scales.

### 3.3. Relationship Between Perceived Sensitivity and Willingness to Share SUD Data

#### 3.3.1. Perceived Sensitivity of Sharing SUD Data and Willingness to Share

Regarding perceived data sensitivity, substance use history had the highest sensitivity score (M = 5.70, SD = 1.64), indicating that participants considered this type of SUD data particularly sensitive. In contrast, substance use medication had the lowest sensitivity score (M = 5.43, SD = 1.86) (Table 3).

The bivariate correlation results showed that the perceived sensitivity of SUD data was negatively correlated with willingness to share data with outside providers (*p* < 0.05), indicating that as participants viewed their SUD-related information as more sensitive, they were less likely to share it. Additionally, higher levels of self-stigma, anticipated stigma, and provider-based stigma were significantly correlated with increased perceptions of data sensitivity (*p* < 0.05).

#### 3.3.2. Stigma and Perceived Sensitivity of Sharing SUD Data

Additionally, all types of stigma (e.g., self-stigma, anticipated stigma, provider-based stigma) were positively correlated with perceived sensitivity of SUD data (ps < 0.001), suggesting that participants who experienced higher levels of stigma were more likely to perceive their SUD data as sensitive (Table 3).

### 3.4. Relationships Between Stigma, Discrimination, Satisfaction, Trust, and “Need to Know” and Willingness to Share

#### 3.4.1. Stigma and Willingness to Share

Self-Stigma and Anticipated Stigma: Participants reported the highest scores for anticipated stigma (M = 3.83, SD = 0.88), followed by self-stigma (M = 3.81, SD = 0.87) and provider-based stigma (M = 2.97, SD = 1.28), indicating that concerns about negative judgments or discrimination from others were prominent (Table 1).

Bivariate correlation analysis (Table 4) showed that self-stigma and anticipated stigma were positively correlated with willingness to share SUD data with providers within the facility (*p* < 0.001). Given that higher scores reflect greater perceived stigma and greater willingness to share, this suggests that participants who internalized stigma (self-stigma) or expected negative judgment from others (anticipated stigma) were more inclined to share their SUD data within their primary care environment.

Provider-Based Stigma: Provider-based stigma was negatively correlated with willingness to share SUD data with providers outside the facility (*p* < 0.001) and emergency providers (*p* < 0.01) (Table 4). Participants who perceived higher levels of stigma from healthcare providers were less likely to share their SUD data with providers beyond their primary facility, including emergency providers.

#### 3.4.2. Provider Discrimination and Willingness to Share

A total of 170 participants (47.6%) reported experiencing discrimination by providers outside the facility, while 31 participants (8.7%) reported discrimination by providers within the facility (Table 1). Individuals reporting discrimination by providers at the facility were significantly less willing to share SUD data with facility providers compared to those who did not report discrimination (*p* < 0.05) (Table 5). Provider discrimination outside the facility was not significantly correlated with willingness to share SUD data with either outside providers (*p* = 0.584) or emergency providers (*p* = 0.065).

#### 3.4.3. Satisfaction and Trust and Willingness to Share

Bivariate correlation results (Table 4) showed that satisfaction with care and trust in providers at the facility were positively correlated with willingness to share SUD data with providers at the facility (ps < 0.001), providers outside of facility (*p* < 0.05, *p* < 0.01), and emergency providers (ps < 0.001).

#### 3.4.4. Need to Know and Willingness to Share

Participants reported a higher willingness score for emergency provider access (4.01 ± 1.32), indicating greater comfort in sharing SUD-related data in emergency situations. Conversely, they reported the lowest willingness score for sharing data with social workers for employment purposes (3.10 ± 1.49), reflecting a higher level of reluctance in this context (Table 2).

“Need to know” scenarios involving outside and emergency providers were positively correlated with willingness to share SUD data with both outside and emergency providers (ps < 0.001). Participants were more willing to share their SUD data when they understood it would be used for specific purposes such as medication and treatment adjustments, improving care, research, emergency situations, employment, and health insurance refunds (ps < 0.001) (Table 6).

#### 3.4.5. Moderation Effects of Trust, Satisfaction, and Gender

The univariate ANOVA results indicated that neither trust nor satisfaction significantly moderated the effects of self-stigma, anticipated stigma, or provider-based stigma on willingness to share across all provider settings (ps > 0.05).

## 4. Discussion

This study is the first to advance the discourse on substance use disorder (SUD) data sharing by centering patient preferences within the broader context of general healthcare. As healthcare systems and policymakers consider changes to 42 CFR Part 2 and seek to refine data-sharing mechanisms, these findings provide critical insights into the ethical, cultural, and systemic factors shaping patient decision-making.

This study also confirmed a two-factor structure (anticipated stigma and self-stigma), supporting the adapted stigma measures’ structural validity in a clinical SUD population. The model fit indices and reliability values indicate that the adapted measures perform comparably or better than the original SASS, reinforcing their robustness in measuring stigma-related constructs among individuals receiving SUD treatment.

The results from this cross-sectional survey of patients at four community-based health clinics reveal a nuanced willingness to share SUD-related information, contingent upon trust, satisfaction with care, and the context or purpose of data sharing. Patients were more likely to share information when the need was clearly defined and relevant to their treatment, aligning with prior studies that highlight the importance of trust and rapport in fostering openness [25]. These findings emphasize the ethical obligation of healthcare providers to build and sustain trusting relationships, particularly in culturally diverse and underserved settings where patients may face additional barriers to care.

Stigma emerged as a critical factor influencing patients’ perceptions of data sensitivity and willingness to share. Consistent with previous research, experiences of self-stigma, anticipated stigma, and provider-based stigma were correlated with heightened concerns about data sharing [26]. However, their effects on willingness to disclose SUD data varied. Self-stigma and anticipated stigma were positively correlated with willingness to share, suggesting that individuals who internalized stigma or expected judgment from others were more likely to disclose their SUD data to providers within their facilities. In contrast, provider-based stigma was negatively correlated with willingness to share, indicating that individuals who perceived discrimination from providers at their facilities were less willing to share their SUD data. These findings underscore the complex influence of stigma on data-sharing behaviors. While self-stigma and anticipated stigma may paradoxically encourage disclosure within familiar and trusted healthcare environments, provider-based stigma heightens fears of discrimination, ultimately discouraging individuals from sharing their SUD data. Stigma continues to be a barrier to delivering best practice SUD care [27,28]. Addressing stigma through provider education and culturally sensitive communication strategies is not only an ethical imperative but also essential for improving health outcomes for populations disproportionately affected by SUD.

Findings point to the need for clear and specific descriptions of what types of data (e.g., SUD history, diagnosis, treatment, labs, and medications) will be shared with whom (i.e., providers within or external to the facility) and under what conditions (i.e., what is the need to know). For example, participants in our study were more willing to share SUD data for purposes directly related to their treatment or health outcomes, such as emergency care or treatment adjustments, but expressed hesitation when the purpose was unrelated, such as for research or administrative tasks. This study highlights the growing demand for granular data-sharing options that allow patients to control what information is shared, with whom, and for what purposes. However, the current capacity to implement such controls in healthcare systems remains limited. While electronic health record (EHR) systems have improved data accessibility overall, most lack the necessary infrastructure to support detailed, patient-specific sharing preferences [28].

### 4.1. Limitations

This study has several limitations that should be considered when interpreting the findings. First, the cross-sectional design prevents the establishment of causal relationships between the variables examined. Second, there is a potential for recruitment bias, as participants who chose to participate in the survey were likely more engaged in their care (e.g., accessing the patient portal) and may not represent the broader population of individuals with SUD.

Additionally, the comprehension of key terms such as “sensitive”/“sensitivity” in the survey may have varied among participants. While survey items provided contextual cues by referencing specific types of SUD-related health information (e.g., substance use history, diagnoses, medications, treatment protocols, and laboratory test results), the term “sensitive” was not explicitly defined. As a result, individual perceptions of data sensitivity may have been influenced by their understanding of the term, which could vary based on factors such as health literacy and personal experiences with SUD care. Future research should provide clear definitions of key terms in survey instruments to ensure consistent interpretation across respondents and minimize variability in understanding.

Finally, data were self-reported, and while participants identified as having substance use problems, no information was collected on formal SUD diagnoses or treatment history, which could influence responses and limit the generalizability of the findings to the full spectrum of individuals with SUD. Fourth, it is important to acknowledge that the analyses were exploratory and that the results should be confirmed in future studies with larger and more diverse samples, including different facility types and geographic regions. Lastly, no information was collected on participants’ SUD history, i.e., duration, severity, substances used, etc.

### 4.2. Conclusions and Implications

This study underscores the ethical and cultural complexities influencing patients’ willingness to share substance use disorder (SUD) data. Decisions around data sharing are not binary but are shaped by interconnected factors, including stigma, trust in providers, satisfaction with care, and the context in which data sharing is requested. These findings highlight the importance of equitable and culturally sensitive approaches to navigating patient preferences in data-sharing practices.

Healthcare providers play an essential role in fostering a supportive environment for SUD data sharing. Building strong, trusting relationships, addressing stigma within clinical settings, and communicating transparently about how sensitive data will be used can empower patients to engage in more open and informed discussions about their care. Such practices not only enhance the patient–provider relationship but also promote dignity, respect, and equity in care delivery.

Current privacy regulations, such as 42 CFR Part 2, provide critical safeguards for patient confidentiality. However, they must evolve to better balance privacy protections with the need for effective care coordination, particularly in culturally diverse and underserved populations. Empowering patients with greater control over how and when their information is shared respects their autonomy, builds trust in healthcare systems, and aligns with the principles of health equity.

The findings also emphasize the need for healthcare systems to enhance electronic health record (EHR) capabilities to support granular privacy controls, improve interoperability, and increase transparency in data-sharing practices. By aligning technology with patients’ preferences and cultural needs, healthcare systems can promote equitable access to care while upholding ethical principles of privacy and autonomy. Future policy revisions and system improvements should prioritize these values to build trust, foster patient-centered care, and ensure that data-sharing frameworks effectively balance the need for information accessibility with the protection of sensitive patient information.

## Figures and Tables

**Table 1:** Descriptive results for all variables (*n* = 357).

Variable	Subcategory	Number	Percentage
Age group	18–29 years old	53	16%
	30–39 years old	107	30%
	40–49 years old	111	31%
	50–59 years old	66	18%
	60 years old or older	20	5%
Income Level	0–10,000 USD	139	39%
	10,001–20,000 USD	92	26%
	20,001–30,000 USD	61	17%
	30,001–40,000 USD	20	6%
	40,001–50,000 USD	15	4%
	Over 50,000 USD	30	8%
Gender	Female	200	56%
	Male	154	43%
	Other	3	1%
Race or Ethnicity	Asian	4	1%
	Black or African American	31	8%
	Hispanic or Latino	93	26%
	More than one race	10	3%
	American Indian or Alaskan Native	11	3%
	Native Hawaiian or Other Pacific Islander	1	1%
	Other	2	1%
	White	205	57%
Maternal Nativity	Foreign	34	10%
	U.S.	323	91%
Education Level	Some High School	57	16%
	High School Graduate (or equivalent)	101	29%
	Some College (1–4 years, no degree)	110	31%
	Associate’s/Technical/Vocational Degree	44	12%
	Bachelor’s Degree (BA, BS, AB, etc.)	33	9%
	Master’s Degree or Higher	7	2%
	Other	5	1%
Provider Discrimination (At Facility)	No	326	91%
	Yes	31	9%
Provider Discrimination (Outside Facility)	No	187	52%
	Yes	170	48%
Variable		Mean	SD
Age in years		41.7	11.2
Annual Income		21,899.6	42,951.6
Self-stigma ^a^		3.8	0.9
Anticipated Stigma ^a^		3.8	0.9
Provider-based Stigma ^a^		3.0	1.3
Satisfaction with Care ^a^		4.1	1.0
Patient Trust in Provider ^a^		4.0	0.8
Willingness at Facility ^a^		4.2	1.12
Willingness outside Facility ^a^		3.2	1.3
Willingness Emergency ^a^		3.8	1.4
Perceived Sensitivity of SUD data ^b^		5.5	1.6
Substance Use History ^b^		5.7	1.6
Substance Use Diagnoses ^b^		5.6	1.8
Substance Use Medication ^b^		5.4	1.9
Substance Use Treatment ^b^		5.4	1.4
Substance Use Lab Tests ^b^		5.5	1.9
Willingness to Share SUD Data all Scenarios (OUTSIDE) ^a^		3.4	1.2
Start/Change Medication ^a^		3.5	1.4
Start/Change no-med Treatment ^a^		3.4	1.4
Improve my Care ^a^		3.6	1.4
Conduct Research ^a^		3.3	1.4
Emergency Provider Access ^a^		4.0	1.3
Social Worker (employment) ^a^		3.1	1.5
Health Insurer (refunds) ^a^		3.2	1.5

Note. ^a^ Potential score range is 1–5. ^b^ Potential score range is 1–7.

**Table 3:** Moderation Effects of Gender on the Relationship Between Sociodemographic Factors and Outcomes.

Source	Dependent Variable	Df	F	*p*
Race * Gender	Patient trust in provider	11	1.92	0.04
	Willingness to share AT	11	2.07	0.02
	Willingness to share Emergency	11	2.22	0.01
Race	Gender	Trust (Mean)	Willingness AT (Mean)	Willingness Emergency (Mean)
White	Women	5.68	5.76	5.8
	Men	5.32	5.4	5.42
Black	Women	5.52	5.61	5.63
	Men	5.37	5.37	5.38
Hispanic	Women	5.64	5.64	5.65
	Men	5.28	5.28	5.3
Asian	Women	6.53	6.53	6.54
	Men	5.67	5.67	5.69
Native American	Women	5.33	5.33	5.35
	Men	5.49	5.49	5.5

Note: The asterisk () indicates an interaction effect. “Race * Gender” represents the interaction between race and gender in predicting the outcome variables.*.

**Table 4:** Bivariate Correlations of Willingness to Share SUD Data with Measures of Stigma, Patient Trust, and Satisfaction (*n* = 357).

Variable	Willingness Within	Willingness Outside	Willingness Emergency	Self-Stigma	Anticipated Stigma	Provider-Based Stigma	Satisfaction	Patient Trust
Willingness Within	-							
Willingness Outside	0.44 **	-						
Willingness Emergency	0.50 **	0.46 **	-					
Self-stigma	0.22 **	0.09	0.07	-				
Anticipated Stigma	0.21 **	0.07	0.09	0.79 **	-			
Provider-based Stigma	−0.18 **	0.07	−0.17 **	0.15 **	0.17 **	-		
Satisfaction	0.44 **	0.16 *	0.23 **	0.32 **	0.27 **	−0.23 **	-	
Patient Trust	0.48 **	0.16 **	0.33 **	0.35 **	0.36 **	−0.32 **	0.82 **	-

Correlation is significant at the 0.01 level. **. Correlation is significant at the 0.05 level.*.

**Table 5:** ANOVA Results (*n* = 357).

Variables	Factors	Sum of Squares	Df	Mean Square	F	*p*-Value
Willingness Within	Provider Discrimination Within	7.62	1	7.62	6.20	0.013
Willingness Outside	Provider Discrimination Outside	0.54	1	0.54	0.30	0.584
Willingness Emergency	Provider Discrimination Outside	6.16	1	6.16	3.42	0.065

**Table 6:** Bivariate Correlations of Willingness to Share SUD Data with Willingness to Share Under All Scenarios (*n* = 357).

Variable	Willingness NTK	Start/Change Medication	Start/Change No-Med Tx	Improve Care	Conduct Research	Emergency Provider	Social Worker	Health Insurer
Willingness NTK	-							
Start/Change Medication	0.83 **	-						
Start/Change No-Med Tx	0.88 **	0.83 **	-					
Improve Care	0.89 **	0.77 **	0.83 **	-				
Conduct Research	0.87 **	0.65 **	0.71 **	0.78 **	-			
Emergency Provider	0.63 **	0.44 **	0.44 **	0.47 **	0.50 **	-		
Social Worker (Employment)	0.78 **	0.49 **	0.59 **	0.60 **	0.65 **	0.38 **	-	
Health Insurer (Refunds)	0.80 **	0.56 **	0.62 **	0.61 **	0.64 **	0.40 **	0.69 **	-

Correlation is significant at the 0.01 level. **.

## Data Availability

The original contributions presented in this study are included in the article/Supplementary Materials. Further inquiries can be directed to the corresponding author(s).

## Supplementary Materials

The following supporting information can be downloaded at: https://www.mdpi.com/article/10.3390/ijerph22040617/s1, Supplementary file S1: survey.
