# Supplementary material for: Balancing Privacy, Trust, and Equity: Patient Perspectives on Substance Use Disorder Data Sharing
Source: Int J Environ Res Public Health. 2025 Apr 15;22(4):617. doi: 10.3390/ijerph22040617 (PMC12027209; doi:10.3390/ijerph22040617)
Supplement: Supplementary file 1 [file ijerph-22-00617-s001.zip › ijerph-3424357-supplementary.pdf]

Survey on Privacy and Protection of Health Records

Questions About Yourself

1. What is your birth year?

   ____

2. What is your gender?
   a. Male
   b. Female
   c. Other ______________________________

3. What is your race or ethnicity? Select all that apply.
   a. Native American or Alaskan native
   b. Asian
   c. Black or African American
   d. Hispanic or Latino
   e. Native Hawaiian or other Pacific Islander
   f. White
   g. Other ____________________________________

4. What is your total yearly income before taxes? (What you make or receive by yourself in a year)

   ____

5. What is your highest level of education?
   a. Some high school
   b. High school graduate (or equivalent)
   c. Some college (1-4 years, no degree)
   d. Associate's degree, technical degree or vocational degree (including occupation or academic degrees)
   e. Bachelor's degree (BA, BS, AB, etc.)
   f. Master's degree or higher
   g. Doctoral degree (PhD, MD, JD, DDS, etc)
   h. Other ______________________________

6. How long have you been receiving substance use treatment at [Facility]?
   a. Less than a month
   b. 1 to 3 months,
   c. 3 to 6 months
   d. 6 to 1 year
   e. More than 1 year
   f. Other ______________________________

Your Experiences with Substance Use and Healthcare

7. Mark the <u>one</u> box that best fits your thoughts and feelings.

**Invisible to Participants**	**Because of my substance use:**	1 Strongly agree	2 Agree	3 Neither agree nor disagree	4 Disagree	5 Strongly disagree
**Self-stigma**	I would feel ashamed.	☐	☐	☐	☐	☐
	I would worry if I could not solve my own problems.	☐	☐	☐	☐	☐
	I would feel weak.	☐	☐	☐	☐	☐
	I would feel like no one would want to get close to me.	☐	☐	☐	☐	☐
	I would feel like a burden to my friends and family.	☐	☐	☐	☐	☐
	I would feel a burden to my colleagues.	☐	☐	☐	☐	☐
**Anticipated stigma**	I would worry other people would think that I am weak.	☐	☐	☐	☐	☐
	I would worry that my friends or family would feel ashamed of me.	☐	☐	☐	☐	☐
	I would worry other people would avoid talking to me.	☐	☐	☐	☐	☐
	I would worry other people would think I was exaggerating my difficulties.	☐	☐	☐	☐	☐
	I would worry other people would think of me as a failure.	☐	☐	☐	☐	☐

	I would worry that other people would say negative things about me.	☐	☐	☐	☐	☐
	I would worry that other people would find out and change how they act toward me.	☐	☐	☐	☐	☐
	I would worry that other people would think I was not capable of recovering.	☐	☐	☐	☐	☐
	I would worry other people would feel sorry for me or patronize me.	☐	☐	☐	☐	☐
**Provider-based stigma**	I would worry that my provider might treat me unfairly compared to those with other medical conditions.	☐	☐	☐	☐	☐
	I would worry that my provider would judge me.	☐	☐	☐	☐	☐
	I would worry that I could not trust my provider with this information.	☐	☐	☐	☐	☐

8. Have you been discriminated against by providers **within** [Facility] because of your substance use?
   a. Yes
   b. No

9. Have you been discriminated against by providers **outside** [Facility] because of your substance use?
   c. Yes
   d. No

10. Mark the <u>one</u> box that best fits your thoughts and feelings about care at [Facility].

		1 Strongly agree	2 Agree	3 Neither agree nor	4 Disagree	5 Strongly

				disagree		disagree
**Patient satisfaction in provider**	I am satisfied with the quality of care I received at [Facility].	☐	☐	☐	☐	☐
	I got the kind of care I wanted at [Facility].	☐	☐	☐	☐	☐
	[Facility] met my needs well.	☐	☐	☐	☐	☐
	I would recommend [Facility] to a friend who was in need of similar help.	☐	☐	☐	☐	☐
	I am satisfied with the amount of help I have received at [Facility].	☐	☐	☐	☐	☐
	The care I received at [Facility] has helped me deal more effectively with my problems.	☐	☐	☐	☐	☐
**Patient trust in provider**	I would feel comfortable discussing my substance use disorder openly with my provider.	☐	☐	☐	☐	☐
	I would be happy to seek help from my provider.	☐	☐	☐	☐	☐
	I would return to [Facility]if I were to seek help again.	☐	☐	☐	☐	☐
	I trust my [Facility]providers decisions about which treatments are best.	☐	☐	☐	☐	☐
	All in all, I completely trust my providers at [Facility]with my care.	☐	☐	☐	☐	☐

Your Willingness to Share Data

11. If asked, how likely are you to share your substance use data with the following types of providers? Choose one box for each provider type.

**Variables/Categories** (Willingness to Share SUD data depending on data recipient)		**1 Always Share**	**2 Often Share**	**3 Sometimes Share**	**4 Rarely Share**	**5 Never Share**
**Health provider within the facility**	Health provider at [Facility].	☐	☐	☐	☐	☐
**Health provider outside the facility**	Health provider outside [Facility].	☐	☐	☐	☐	☐
**Emergency health provider**	Emergency health provider.	☐	☐	☐	☐	☐

12. Do you consider any of the following types of data to be sensitive? Choose one box for each data type.

**Variables/ Categories**		**1 Extremely Sensitive**	**2 Very Sensitive**	**3 Moderately Sensitive**	**4 Somewhat Sensitive**	**5 Slightly Sensitive**	**6 Barely Sensitive**	**7 Not at all Sensitive**
**Sensitivity of sharing substance use-related data**	Substance use history.	☐	☐	☐	☐	☐	☐	☐
	Substance use diagnoses.	☐	☐	☐	☐	☐	☐	☐
	Substance use medication.	☐	☐	☐	☐	☐	☐	☐
	Substance use treatment.	☐	☐	☐	☐	☐	☐	☐
	Substance use laboratory tests.	☐	☐	☐	☐	☐	☐	☐

13. If asked, how likely are you to share your substance use data **outside** [Facility]under the following conditions? Choose <u>one</u> box for each condition.

**Variables/ Categories** (Willingness to share SUD data depending on data sharing purpose/ "need to know")		**1 Always Share**	**2 Often Share**	**3 Sometimes Share**	**4 Rarely Share**	**5 Never Share**
**Medication**	My provider outside [Facility]wants to start or change a new medication.	☐	☐	☐	☐	☐
**Treatment**	My provider outside [Facility]wants to start or change a non-medication the treatment.	☐	☐	☐	☐	☐
**Better Care**	My provider outside [Facility]wants to improve my care.	☐	☐	☐	☐	☐
**Research**	My provider outside [Facility]wants to conduct research.	☐	☐	☐	☐	☐
**Emergency**	My emergency provider outside [Facility]wants to look at my data in a life-threatening situation.	☐	☐	☐	☐	☐
**Employment**	My social worker outisde [Facility]wants to discuss my employment.	☐	☐	☐	☐	☐
**Health insurance**	My health insurer wants to work on the refunds.	☐	☐	☐	☐	☐
